# Imported malaria cases in former endemic and non-malaria endemic areas in China: are there differences in case profile and time to response?

**DOI:** 10.1186/s40249-019-0571-3

**Published:** 2019-07-05

**Authors:** Shao-Sen Zhang, Jun Feng, Li Zhang, Xiang Ren, Elizabeth Geoffroy, Sylvie Manguin, Roger Frutos, Shui-Sen Zhou

**Affiliations:** 1 National Institute of Parasitic Diseases, Chinese Center for Disease Control and Prevention; Chinese Center for Tropical Diseases Research; Key Laboratory of Parasite and Vector Biology, Ministry of Health; National Center for International Research on Tropical Diseases, Ministry of Science and Technology; WHO Collaborating Center for Tropical Diseases, Shanghai, China; 2 HydroSciences Montpellier (HSM), Institut de Recherche pour le Développement (IRD), CNRS, Université de Montpellier, 34093 Montpellier, France; 3 IES Université de Montpellier, CNRS, 34059 Montpellier Cedex 5, France; 4 Cirad, UMR 17, Intertryp, Campus international de Baillarguet, 34398 Montpellier Cedex 5, France; 5 Division of Infectious Diseases, Key Laboratory of Surveillance and Early-warning on Infectious Disease, Chinese Center for Disease Control and Prevention, Beijing, China; 6 Global AIDS Interfaith Alliance, San Rafael, California, USA

**Keywords:** Imported malaria, Non-malaria endemic area, Malaria endemic area, Surveillance and response, Health facilities performance

## Abstract

**Background:**

China has achieved zero indigenous malaria case report in 2017. However, along with the increasing of international cooperation development, there is an increasing number of imported malaria cases from Chinese nationals returning from malaria-affected countries. Previous studies have focused on malaria endemic areas in China. There is thus limited information on non-endemic areas in China, especially on the performance of malaria surveillance and response in health facilities.

**Methods:**

A comparative retrospective study was carried out based on routine malaria surveillance data collected from 2013 to 2017. All imported malaria cases reported within the mainland of China were included. Variables used in the comparative analysis between cases in former endemic and former non-endemic areas, included age, gender and occupation, destination of overseas travel, *Plasmodium* species and patient health outcome. Monthly aggregated data was used to compare seasonal and spatial characteristics. Geographical distribution and spatial-temporal aggregation analyses were conducted. Time to diagnosis and report, method of diagnosis, and level of reporting/diagnosing health facilities were used to assess performance of health facilities.

**Results:**

A total of 16 733 malaria cases, out of which 90 were fatal, were recorded in 31 provinces. The majority of cases (96.2%) were reported from former malaria endemic areas while 3.8% were reported from former non-malaria endemic areas. Patients in the age class from 19 to 59 years and males made the highest proportion of cases in both areas. There were significant differences between occupational categories in the two areas (*P* <  0.001). In former endemic areas, the largest proportion of cases was among outdoor workers (80%). Two peaks (June, January) and three peaks (June, September and January) were found in former endemic and former non-endemic areas, respectively. Time between the onset of symptoms and diagnosis at clinics was significantly different between the two areas at different level of health facilities (*P* <  0.05).

**Conclusions:**

All the former non-endemic areas are now reporting imported malaria cases. However, the largest proportion of imported cases is still reported from former endemic areas. Health facilities in former endemic areas outperformed those in former non-endemic areas. Information, treatment, and surveillance must be provided for expatriates while capacity building and continuous training must be implemented at health facilities in China.

**Electronic supplementary material:**

The online version of this article (10.1186/s40249-019-0571-3) contains supplementary material, which is available to authorized users.

## Multilingual abstracts

Please see Additional file 1 for translations of the abstract into the five official working languages of the United Nations.

## Background

According to the 2018 World Malaria Report, 219 million malaria cases and 435 000 associated deaths were reported globally in 2017 [1]. Malaria control efforts across China have led to the decrease of both morbidity and mortality over the past 60 years, from about 30 million cases each year in 1950 to about 7000 cases in 2010 [2–8]. Following implementation of the National Malaria Elimination Program (NMEP) in 2010, which aims to eliminate local transmission by 2020, local malaria transmission steadily declined throughout the country and achieved the goal of zero indigenous malaria case report in 2017 [2, 5, 6, 9]. By contrast, the number of malaria cases reported around the world consistently increased to 219 million in 2017 from 216 million in 2016 and 212 million in 2015. The rise in malaria morbidity in African and South-East Asian countries is substantial with countries displaying more than 20% increase [1, 10–12].

Within the mainland China, thousands of imported cases are still reported every year with a minimal decline in the past 5 years [6, 7, 13, 14]. These cases clearly pose a risk of re-introduction with important public health implications highlighted by policy makers and researchers [5, 13, 15, 16]. With the launch of the Belt and Road Initiative in 2013, international cooperation and international travel of Chinese nationals to malaria-affected countries, particularly in sub-Saharan Africa have increased [13, 15]. The seasonal characteristics of the imported cases differ from indigenous cases [13, 15] while the geographic distribution has also changed as imported malaria cases are now occurring in both former endemic and former non-endemic areas. Furthermore, the species of *Plasmodium* involved have shifted from only *Plasmodium falciparum* and *P. vivax* for the previous locally transmitted cases to four human *Plasmodium* spp. (including *P. malariae* and *P. ovale*) among imported cases [13].

Prior to 2015, studies only focused on the global national performance of health facilities or of those in former endemic areas only [17]. However, some preliminary studies have found significant differences on the malaria diagnosis capacities within China between health facilities in former malaria endemic and former non-endemic areas [18, 19]. Health workers in former malaria endemic areas had better knowledge of malaria epidemiology and malaria diagnosis than those from former non-endemic areas [19, 20]. Misdiagnosis of malaria cases may delay appropriate treatment and negatively impact health outcomes, and may lead to re-introduction of malaria, undermining the progress made through the malaria elimination campaign [21, 22]. These issues warrant an investigation on the characteristics of imported malaria cases and the performance of the health system. This study thus aims at comparing the profile of malaria cases reported in China from 2013 to 2017, time to response and capacity of response of health facilities in former endemic and former non-endemic areas.

## Material and methods

### Definitions

#### Former endemic areas

Historically, 24 provinces in mainland China were considered as malaria endemic areas with suitable environmental conditions for malaria vectors and local malaria transmission [23].

#### Non-endemic areas

The areas display no suitable environmental conditions for malaria vector breeding and no local transmission of malaria was previously reported. The requirement for surveillance and response to malaria cases at county and township level was different in former endemic and former non-endemic areas [17, 23].

#### Imported cases

According to the WHO malaria terminology, an imported case corresponds to a patient who acquired malaria infection outside the area where it is diagnosed [24]. Since there is no routine laboratory test to identify an “imported” case, the determination is achieved by investigation of patients’ travel history to malaria endemic areas through epidemiological survey.

### Data source and data collection

Variables used in the comparison of the demographic characteristics of reported imported malaria cases between former endemic and former non-endemic areas included the following: age, gender, occupation, destination of overseas travel, *Plasmodium* species and patient health outcome. To compare the seasonal and spatial characteristics of imported malaria cases from 2013 to 2017, their number was aggregated by month and plotted based on area classification. Finally, to compare the performance of malaria case identification and diagnosis, we created two duration variables using date of onset, date of diagnosis and date of report, together with other variables reflecting the method of diagnosis and the level of the reporting/diagnosis health facility for each case. According to the Chinese Law on Prevention and Treatment of Infectious Diseases (CLPTID) and to International Heath Regulation (IHR), malaria is a notifiable infectious disease. Health facilities at each of the administrative level are required to report every case within 24 h after diagnosis to the Chinese Infectious Disease Report System (CIDRS), a web-based reporting system for individual cases and data management for notifiable infectious diseases. All imported malaria cases reported in CIDRS between 2013 and 2017 from all the health facilities within the mainland of China (excluding Hong Kong, Macau and Taiwan) were included in the analysis. Information on individual cases in this study was obtained from CIDRS, which includes general demographic data, diagnosis data, treatment data and epidemiological data. Data used for this study was routinely collected as part of NEMP from 2013 to 2017.

#### Geographic and statistical analysis

The geographical distribution and the spatial-temporal aggregation analysis were performed using ArcGIS 10.0 (Esri Inc., Redlands, CA, USA). The comparative analysis between variables from former malaria endemic and former non-endemic areas was conducted with *t* tests and *Chi-square* tests using SPSS (version 25, IBM Corp, Armonk, NY, USA). The level of significance was set at *P* <  0.05.

## Results

### Demographic characteristics

A total of 16 733 malaria cases were reported from 31 provinces in the mainland China from 2013 to 2017 with 90 (0.54%) related deaths. Demographic and geographic characteristics of the imported malaria cases are shown in Table 1. The majority of cases, *n* = 16 090 (96.2%), were reported from former malaria endemic areas while 643 (3.8%) cases were reported from former non-endemic areas. The age group ranging from 19 to 59 years and males made the overwhelming proportion of cases in both former endemic and former non-endemic areas (Table 1). There were significant differences between occupational categories of imported malaria cases in former endemic and former non-endemic areas (*P* <  0.001). In former endemic areas, the largest proportion of cases were outdoor workers (80%), with indoor workers making up to 10% of cases while the final 10% were unclearly identified (Table 1). Conversely, cases recorded in former non-endemic areas corresponded to indoor workers (39%) more than to outdoor workers (29%) while the occupation of 32% of cases was undetermined.

**Table 1:** Demographic characteristics of imported malaria cases in China, 2013–2017

Demographic Characteristics	Number of cases reported				*P* value
	Former malaria endemic areas		Non malaria endemic areas		
	Number	Proportion (%)	Number	Proportion (%)	
Total Cases	16 090		643		
Age					0.8
< 5 years	62	< 1	0	0	
5–18 years	240	1	6	1	
19–59 years	15 569	97	623	97	
≥ 60 years	219	1	12	2	
Gender					0.2
Male	15 172	94	598	93	
Female	918	6	45	7	
Occupation					< 0.001
Outdoor workers^a^	12 370	80	180	29	
Indoor workers^b^	1613	10	246	39	
Unclear^c^	1532	10	197	32	
Missing^d^	575	–	20	–	
Destination of overseas travel					< 0.001
Africa	12 436	80	475	94	
Southeast Asia/South Asia	3011	19	16	3	
South America	24	0	0	0	
Oceania	137	1	15	3	
Other: West/East Asia	4	0	0	0	
Missing^d^	478	–	137	–	
*Plasmodium* species					< 0.001
*P. vivax*	3928	24	64	10	
*P. falciparum*	10 278	64	481	75	
*P. malariae*	300	2	11	2	
*P. ovale*	1297	8	16	2	
*P. falciparum + P. ovale*	105	1	1	0	
*P. falciparum + P. vivax*	146	1	3	0	
Undiagnosed/missing	36	0	67	10	
Fatal outcome					
Death reported	76	0	14	2	–

-: Not applicable

^a^Outdoor workers: persons whose activity is mostly conducted outside. This includes Architectural engineers, Construction workers, Farmers, Fishermen, Overseas migrant worker (Expatriate Chinese nationals), Open mine workers, Sailors/Truck drivers, Field engineers, Herdsmen, Militaries/Soldiers, etc.

^b^Indoor workers: work mostly indoor, including: Businessmen, Caterers, Interpreters, Medical staff, Office workers, Teachers, Actors, Flight attendants, Baby-sitters, Middlemen, Cooks, Diplomats, Financial staff, Journalists, Underground mine workers, Prisoners (although not a “worker” per se, a prisoner is officially classified as an indoor worker since his/her time is spent indoor), Researchers, Waiters, etc.

^c^Unclear: the risk exposure cannot clearly be estimated. Children, Retirees, self-employees, Students, Unemployed people, Sportsmen and Sportswomen, Tourists, etc.

^d^missing data were not included into statistical analysis

### Epidemiological characteristics

**Fig. 1:**
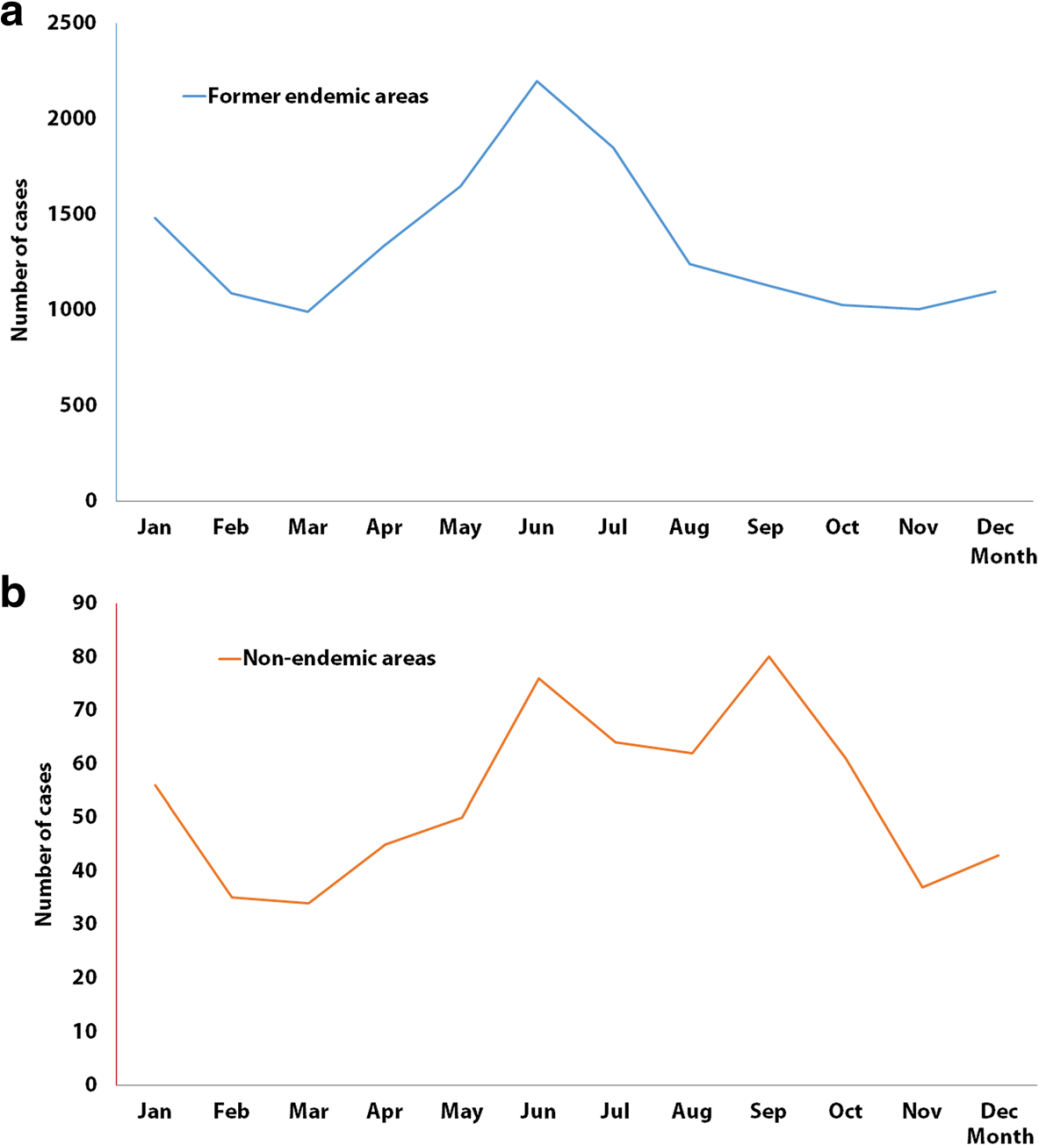
Seasonal dynamic of imported malaria cases in former endemic and non-endemic areas in China, aggregated 2013–2017. **a** Seasonal dynamic in former endemic areas. **b** Seasonal dynamic in non-endemic areas

**Fig. 2:**
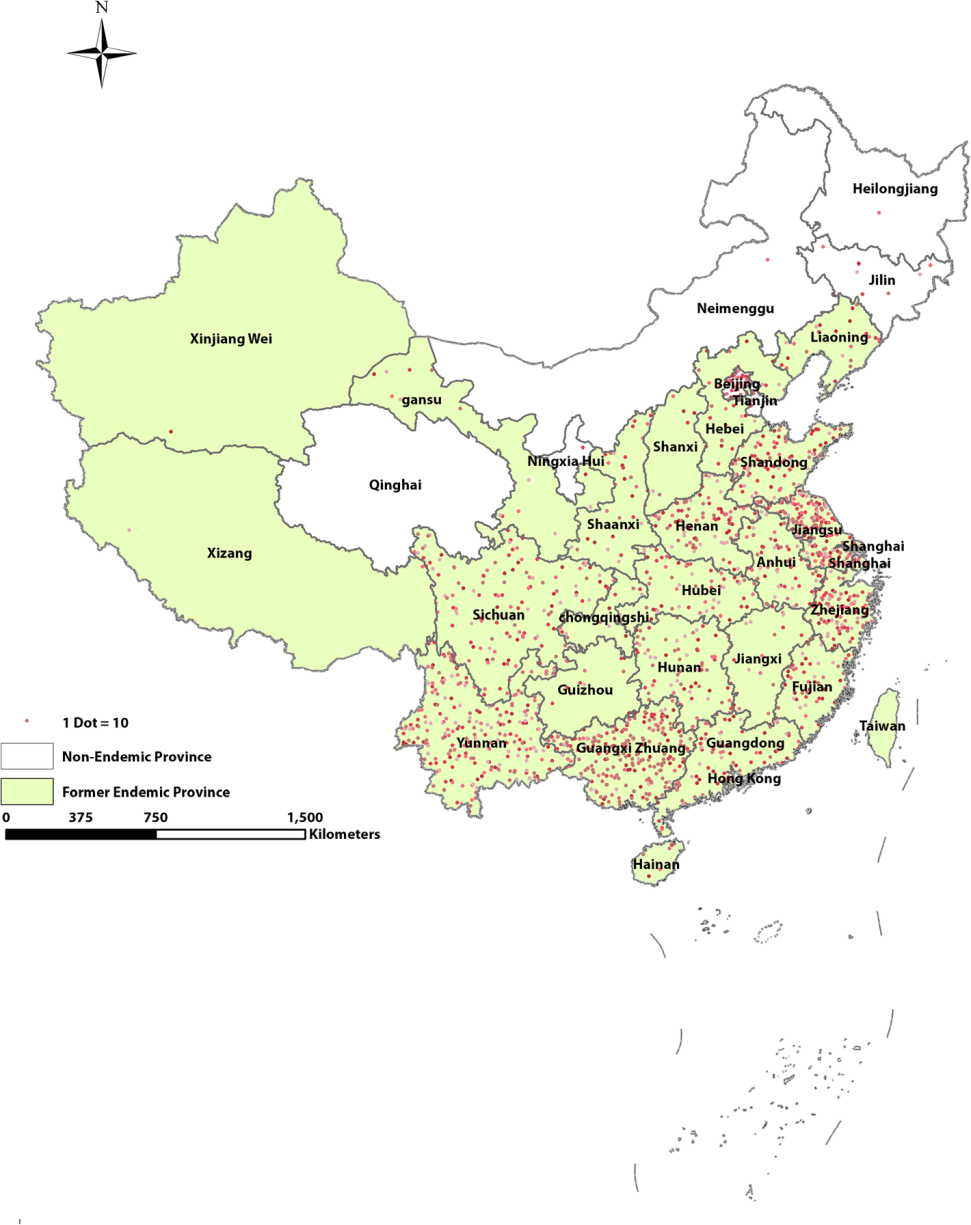
Geographic distribution of imported malaria cases in former endemic and non-endemic areas in China, 2013–2017

Two peaks, i.e. June and January (Fig. 1a) and three peaks, i.e. June, September and January (Fig. 1b) were observed in former endemic and former non-endemic areas, respectively. Imported cases in former endemic areas were clustered in the Eastern coastal region and in the Southwestern border area, whereas cases were scattered in former non-endemic areas (Fig. 2). The destination of overseas travel of imported cases reported from former endemic and former non-endemic areas were found to be significantly different (*P* <  0.001). The imported cases reported in former non-endemic areas were primarily coming from Africa (94%), while a significant number of cases reported in former endemic areas were from Southeast Asia (19%) in addition to Africa (80%) (Table 1). Few cases were from Oceania in both endemic (1%) and former non-endemic areas (3%). With respect to the *Plasmodium* species, *P. falciparum* (75%) was the predominant species in former non-endemic areas, whereas there was a larger proportion of *P. vivax* in former endemic areas (*P. vivax* 24%, *P. falciparum* 64%). The proportion of *P. malariae* was almost the same in former non-endemic and former endemic areas (2%), while a larger proportion of *P. ovale* was reported in former endemic areas (8%) than in former non-endemic areas (2%). More cases were reported as “undiagnosed/missing diagnosis information” in former non-endemic areas than in former endemic areas (10% vs 0%) (Table 1).

### Performance of health facilities

The duration between onset and diagnosis at admission was significantly different both between former endemic and former non-endemic areas and between different levels of health facilities (*P* <  0.05) (Table 2). The significant difference between health facilities in former endemic and former non-endemic areas was found in the time required from case diagnosis to case reporting (*P* <  0.001) (Table 2). No significant difference was found in the method of diagnosis between former endemic and former non-endemic areas. Nearly all cases were laboratory confirmed (99.6% in former endemic and 95.8% in former non-endemic). However, the majority of cases were diagnosed at the prefecture level (68%) in former non-endemic areas whereas in former endemic areas 41% of cases were diagnosed at the county level and 45% at the prefecture level (Table 3).

**Table 2:** Duration between onset of malaria symptoms and diagnosis and diagnosis and reporting in China, 2013–2017

	Number of cases	Former malaria endemic areas		Number of cases	Non malaria endemic areas		*P* value
		Median	IQR^a^		Median	IQR^a^	
Time from onset to diagnosis							
Total	15 965	3.54	(1.63–6.63)	643	4.42	(2.42–10.46)	0.03
Township	1023	2.75	(1.67–4.67)	1	0.42	(0.42–0.42)	
County	6568	2.71	(1.42–5.46)	20	1.96	(0.74–5.21)	
Prefecture and above	7270	4.38	(2.33–7.83)	438	4.42	(2.46–9.47)	
Private hospital	232	3.48	(1.59–5.53)	4	3.58	(2.7–6.06)	
Provincial	804	4.48	(2.38–8.66)	177	4.63	(2.44–13.46)	
POE^b^	68	2.65	(0.64–5.67)	3	1.58	–	
Time from diagnosis to report							
Total	15 965	0.05	(0.00–0.5)	643	0.07	(0.00–0.73)	
Township	1023	0.06	(−0.46–0.25)	1	0.96	(0.96–0.96)	
County	6568	0.07	(0.00–0.58)	20	−0.4	(− 0.58–0.13)	
Prefecture and above	7270	0.05	(−0.46–0.56)	438	0.49	(0.04–0.92)	
Private hospital	232	0.16	(0.05–0.69)	4	0.37	(−0.52–0.84)	
Provincial	804	−0.5	(−0.67–0.33)	177	−0.54	(− 0.63–-0.38)	
POE^b^	68	−0.5	(−0.63–0.42)	3	−0.58	–	

-Not applicable

^a^IQR: Interquartile range;

^b^POE: Point of Entry at the customs

**Table 3:** Comparison between method of diagnosis and level of reporting/diagnosis facility, China, 2013–2017

	Former malaria endemic areas				*P* value
	Number	Proportion (%)	Number	Proportion (%)	
Method of diagnosis					0.9
Laboratory confirmed^a^	16 021	99.6	616	95.8	
Clinical	69	0.4	27	4.2	
Level of reporting & diagnosis health facility					< 0.001
Township	1023	6	1	< 1	
County	6568	41	20	3	
Prefecture	7270	45	438	68	
Private hospital	232	1	4	< 1	
Provincial	804	5	177	27.5	
POE^b^	68	< 1	3	< 1	
Missing	125	< 1	0	0	

^a^Diagnosis confirmed by Laboratory test which include Rapid Diagnosis Test (RDT), Polymerase Chain Reaction (PCR), Microscopy

^b^POE: Point of entry, screen test at customs;

## Discussion

The main feature in this analysis is the overwhelming presence of Africa as a travel destination among patients infected with malaria. Travelers to Africa represent 80% of patients from former endemic areas and 94% of patients from former non-endemic areas. The top ten African countries found as the original infection of these imported cases were Angola, Nigeria, Democratic of Republic Congo, Chad, Uganda, Equatorial Guinea, Guinea, Cameroon, Sudan and Tanzania. Patients are almost exclusively men in the professionally-active class of age (19 to 59 years). The vast majority of patients are Chinese nationals went abroad to work on international projects and coming back home. This reflects the international involvement of Chinese companies in Africa. The overwhelming presence of this socio-professional class among malaria patients also matches the location of cases in major cities from the East coast [13, 15, 25]. Indeed, this correlates with the presence of air transportation hubs and labor export companies mainly in major cities on the East coast (http://femhzs.mofcom.gov.cn/fecpmvc/pages/fem/corp_ml_list2.jsp). Travel patterns, air network distribution, trade connection and malaria situation in the visited countries are features commonly considered to influence the risk of malaria introduction [26–28]. China is a country with a history of malaria endemicity who is now on the way to malaria elimination. Environmental conditions and efficient vectors are thus present and the risk of reestablishment following introduction is possible.

This study shows that travel to Africa for work may be the most important driver of imported malaria within China and the biggest risk for re-introduction. However, comparing former endemic and former non-endemic areas in China provides a more detailed view of the dynamic. A different pattern is observed between former endemic and former non-endemic areas. In former non-endemic areas, the introduction is due almost exclusively to workers coming back from Africa but the cases are equally distributed between outdoor workers and indoor workers. Indoor workers are not likely to be exposed to malaria vectors, which have a nocturnal behavior, during indoor day-time working hours. Nevertheless, they are as much affected as outdoor workers during the night time. The main cause of infection seems therefore to be the long presence in an endemic country and exposure to malaria vectors during everyday life, especially at night, rather than exposure due to occupation which occurs at day, and indoors for half of the reported cases. This also makes sense considering that malaria vectors are mostly nocturnal mosquitoes when occupations usually occur at day time. In former endemic areas there is a high concentration of outdoor workers (80%). There is no environmental reason to explain this difference. Workers from former endemic areas are exposed to the same conditions in Africa as workers from former non-endemic areas. An explanation might be that the typology of work for travelers differs between those coming from former endemic and former non-endemic areas, more outdoor workers coming from the former and more inside workers coming from the latter. Another main difference can be observed. In former non-endemic areas patients are lmost exclusively traveling to Africa (94%) whereas in former endemic areas only 80% are working in Africa and 19% are working in Southeast/South Asia. The most plausible reason for this difference is that some of former endemic areas are located along the Southern Chinese border and have thus established partnership with Southeast/South Asian countries with a tradition of expatriate workers and cross-border movements of populations [29–31]. It is also very likely that the typology of work might be more oriented in these former endemic areas towards outside occupations. The difference in the peaks of malaria observed between the two kinds of areas in China might also be related to this difference in proximity and to different patterns of the migrant population, such as the frequency of labor dispatching, holiday celebrations, local farming system, etc. [15, 25, 32–34]. The additional peak in September–October in former endemic areas can thus be attributed to the easier conditions of traveling from Southeast/South Asia. African countries are a lot more distant making traveling more difficult and expensive and rotations clearly defined in duration.

Another main difference observed between former endemic areas and former non-endemic areas is the efficiency of reaction of health facilitates when admitting a case of malaria. The differing performance of health facilities in reporting and diagnosing malaria between former endemic and former non-endemic areas and depending on heath facility levels is clearly highlighting the need for strengthening the training of staff in malaria case detection, diagnosis and treatment. Fast detection and reporting were performed equally efficiently in health centers at the township/county and prefecture levels in former endemic areas whereas this achievement was encountered only at the prefecture level in former non-endemic areas. This might well be a consequence of the NMEP strategy to focus on capacity building towards county and community level facilities in former endemic areas. The capacity of malaria diagnosis and treatment in health facilities are key factors to efficiently implement detection, surveillance and response, especially at malaria elimination stage [35, 36]. Timely case detection and treatment will help to prevent the re-introduction of malaria in former endemic areas and reduce the occurrence of fatal issues [15, 27, 37]. There is thus an urgent need of intensive capacity building and training for the township/county health centers. Nevertheless, continuous capacity building must be implemented in former endemic areas in order to maintain the level of competence.

With the development of international cooperation, exemplified by the Belt and Road Initiatives, the main source of malaria infection and the main risk for malaria elimination are linked essentially to expatriate workers coming back from Africa and to a lower extent from Southeast/South Asia. This risk must be tackled at two levels. At the upstream level, there is a clear need to better equip expatriates with malaria prevention information and tools, such as risk exposure prevention, information on common symptoms, treatment options, before travelling to malaria endemic areas. This must be completed with the availability of appropriate antimalaria drugs [38]. A last aspect to consider at this level is the establishment of detection centers and detection campaigns on site in Africa by the companies employing expatriate workers. This should be preferably extended also to Southeast/South Asian countries. At the downstream level, there is a need for intensive and continuous capacity building for health centers in order to maintain the capacity of fast detection, an essential element for managing the risk of malaria introduction [36, 39].

There are limitations to this study relating to data quality and availability of data. Data availability was dependent on the recording by health facility staff. Missing data and unclear coding made up to 3% of occupation data. Detailed information on movements of populations, i.e. travel frequency, purpose of travel, etc., was not recorded. Standardized forms should thus be developed in order to record additional. However, this study was important as it addressed the situation of imported malaria and the health system performance in former endemic areas but more importantly in former non-endemic areas in China, which was rarely conducted before, previous works focusing mostly on endemic areas. Additionally, researchers adhered to the Strengthening the Reporting of Observational Studies in Epidemiology (STROBE) guidelines for reporting on observational research and the Reporting of studies Conducted using Observational Routinely-collected health Data (RECORD) statement for studies using routinely collected programmatic data [40, 41].

China has achieved zero indigenous case report in 2017 and is on the way to eliminate malaria by 2020 as planned [23]. However, together with the open-up policy and increase of international cooperation, imported malaria cases are now commonly reported across the country [8, 13, 15]. Further studies should therefore focus on the cross-border transmission, surveillance and response in major cities with detailed social and economic data. These studies should bring recommendations for proper control in areas massively affected by imported malaria.

## Conclusions

Imported malaria was found to be more widely distributed in China from 2013 to 2017 than expected. All former non-endemic areas are now reporting imported malaria cases. However, the largest proportion of reports of imported cases is still coming from former endemic areas. The demographic characteristics of imported malaria depends upon the country of expatriation, species composition of parasites, occupation and place of origin of workers. Health facilities in former endemic areas outperformed those in former non-endemic areas, suggesting that targeted training for health staff in former non-endemic areas should be a priority along with proper information of expatriates and availability of drugs and detection on site in foreign countries.

## Additional file

Additional file 1: Multilingual abstracts in the five official working languages of the United Nations. (PDF 448 kb)

## Data Availability

The datasets used and/or analyzed during the current study are available from the corresponding author on reasonable request.

## Abbreviations

CIDRS: Chinese Infectious Disease Report System
CLPTID: Chinese Law on Prevention and Treatment of Infectious Diseases
IHR: International Heath Regulation
NMEP: National Malaria Elimination Program
RECORD: The REporting of studies Conducted using Observational Routinely collected health Data
STROBE: Strengthening the Reporting of Observational Studies in Epidemiology
