# Supplementary material for: Imported malaria cases in former endemic and non-malaria endemic areas in China: are there differences in case profile and time to response?
Source: Infect Dis Poverty. 2019 Jul 5;8:61. doi: 10.1186/s40249-019-0571-3 (PMC6610923; doi:10.1186/s40249-019-0571-3)
Supplement: Supplementary file 1 — Multilingual abstracts in the five official working languages of the United Nations. (PDF 448 kb) [file 40249_2019_571_MOESM1_ESM.pdf]

Translation of the abstract into the five official working languages of the United Nations

حالات الملاريا المستوردة في المناطق المستوطنة سابقاً والغير مستوطنة بالملاريا في الصين: هل هناك اختلافات في ملف الحالات ووقت الاستجابة؟

المؤلف: شاوزن زانج، جان فينج، لي زانج، زياتج رين، اليزابيث جوفيري، سيلفي مونجان، روجيه فروتوس، شيزين زاو

ملخص

تمهيد: في عام 2017، لم تسجل الصين أي حالة من السكان الاصلين لمرض الملاريا. ومع ذلك، إلى جانب سياسة الانفتاح وتنمية التعاون الدولي، هناك عدد متصاعد من حالات الإصابة بالملاريا المجلوبة من المواطنين الصينيين العائدين من البلدان المتأثرة بالملاريا. ركزت الدراسات السابقة على المناطق المستوطنة بالملاريا في الصين. وبالتالي، هناك معلومات محدودة عن المناطق غير الموبوءة في الصين، وخصوصاً عن أداء مراقبة الملاريا والاستجابة لها في المرافق الصحية.

المنهج: تم إجراء دراسة مقارنة بأثر رجعي بناءً على بيانات مراقبة الملاريا الروتينية التي تم جمعها من عام 2013 إلى 2017. وشملت الدراسة جميع حالات الملاريا المستوردة المبلغ عنها داخل الصين. المتغيرات المستخدمة في التحليل المقارن بين الحالات في المناطق المستوطنة سابقاً والمناطق غير المستوطنة، وتشمل: العمر والجنس والمهنة، وجهة السفر إلى الخارج، وأنواع البلازموديوم ونتائج صحة المريض. واستخدمت البيانات المجمعة الشهرية لمقارنة الخصائص الموسمية والمكانية. وأجريت تحليلات للتوزيع الجغرافي والتجميع المكاني-الزمني. وقد استخدم الوقت اللازم للتشخيص والإبلاغ، وطريقة التشخيص، ومستوى الإبلاغ/تشخيص المرافق الصحية لتقييم أدائها.

النتائج: تضمن هذه الدراسة 16733 حالة إصابة بالملاريا، منها 90 حالة مميتة في 312 مقاطعة في الصين. تم الإبلاغ عن غالبية الحالات 96.2٪ من المناطق المستوطنة سابقاً بالملاريا بينما تم الإبلاغ عن 3.8 ٪ من المناطق الغير مستوطنة بالملاريا. وشكل الذكور والمرضى في الفئة العمرية بين 19 إلى 59 سنة أعلى نسبة من الحالات في كلتا المنطقتين. وكانت هناك فروق كبيرة بين الفئات المهنية في كلتا المنطقتين ($p < 0.001$). وكانت أكبر نسبة من الحالات في المناطق المستوطنة سابقاً بين العاملين في الهواء الطلق (80%). يوجد اعلى قمتين في أشهر (يونيو ويناير) في المناطق المستوطنة سابقاً، اما عن المناطق الغير مستوطنة سابقاً فيوجد ثلاث قمم في أشهر (يونيو، سبتمبر ويناير). وكان الوقت بين بداية الأعراض والتشخيص في العيادات مختلفا اختلافا كبيرا بين المنطقتين على مستوى مختلف من المرافق الصحية ($p < 0.05$).

الاستنتاجات: جميع المناطق غير المستوطنة تبلغ عن حالات الإصابة الملاريا المستوردة. ومع ذلك، أن أكبر نسبة من الإبلاغ عن الحالات المستوردة لا تزال تأتي من المناطق المستوطنة سابقا. تفوق أداء المرافق الصحية في المناطق المستوطنة سابقاً أداء المرافق الصحية في المناطق غير المستوطنة. ويجب توفير المعلومات والعلاج والمراقبة للمغتربين بينما يجب تنفيذ بناء القدرات والتدريب المستمر الدائم في المرافق الصحية في الصين.

الكلمات الرئيسية: الملاريا المستوردة، المناطق غير المستوطنة بالملاريا، المناطق المستوطنة بالملاريا، المراقبة والاستجابة، أداء المنشآت الصحية.

Translated from English version into Arabic by Dr. Aly Shamseddin

中国疟疾流行区和非流行区输入性疟疾病例数据分析

张少森, 丰俊, 张丽, 任翔, Elizabeth Geoffroy, Sylvie Manguin, Roger Frutos, 周水森

摘要

引言：中国于 2017 年实现疟疾本地感染病例零报告。然而，随着改革开放和国际合作不断推进，由中国赴境外疟疾流行区务工人员增加所致的输入性疟疾也呈逐年上升的态势。 既往的相关研究多关注中国国内疟疾流行区报告病例的情况，较少关注非流行区，特别是医疗机构在疟疾病例监测与响应的情况。
方法：本研究基于 2013-2017 年全国疟疾病例监测系统报告的输入性疟疾病例开展回顾性比较分析。数据分析变量包括病例的年龄、性别、职业、出国居留地、疟原虫种类和预后（是否治愈或死亡）。 通过基于月度汇总病例数，开展空间和时空回归模型分析，获得输入性疟疾病例的季节特征和空间分布特征在疟疾流行区和非流行区的差异。基于分析病例确诊时间和报告时间的间隔，诊断方式，病例确诊和报告的医疗机构级别等指标差异，评估两类地区的医疗机构在输入病例监测与响应能力的差异。
结果：2013-2017 年全国 31 个省级行政区共报告输入性疟疾 16733 例，其中 90 例死亡病例。疟疾流行区的医疗机构报告了大部分的病例(占 96.2%)，非疟疾流行区报告病例占 3.8%。年龄构成中，19-59 岁年龄组在两类地区中均占大多数。 职业构成在两类地区差异具有统计学意义 $P<0.001$：在疟疾流行区报告的输入性疟疾病例中户外工作人员占 80%。 病例的季节分布中，在疟疾流行区分别在一月份和六月份出现两个高峰，而疟疾非流行区则在一月、六月和九月出现三个高峰。输入性疟疾病例的发病时间和确诊时间间隔在两类区域和不同级别的医疗机构间存在差异，且差异具有统计学意义 ($P < 0.05$)。
结论：尽管所有的疟疾非流行地区均有输入性疟疾病例报告，但大部分的输入性疟疾病例仍然出现在疟疾流行区。疟疾流行区的医疗机构在输入性疟疾的诊治方面表现好于疟疾非流行区的医疗机构。因此，应针对医疗机构的工作人员开展持续性的疟疾诊治、监测等相关培训。

Translated from English version into Chinese by Mr. Shaosen Zhang

Paludisme importé en Chine dans les régions anciennement endémiques et non endémiques : différences de profil et de délai de réponse ?

Auteurs : Shaosen Zhang, Jun Feng, Li Zhang, Xiang Ren, Elizabeth Geoffroy, Sylvie Manguin, Roger Frutos, Shuisen Zhou

Résumé

Contexte : La Chine n'a enregistré aucun cas autochtone de paludisme depuis 2017. Cependant, suite à la politique d'ouverture et au développement de la coopération internationale, le nombre de cas de paludisme importés par des ressortissants chinois revenant de pays impaludés est en augmentation. Les études antérieures ont porté essentiellement sur les régions chinoises impaludées et il existe donc peu d'information sur les zones initialement non endémiques ainsi que sur les performances du système de santé dans ces régions.
Méthodes : Une étude rétrospective comparative a été réalisée à partir des données de surveillance du paludisme recueillies de 2013 à 2017. Tous les cas de paludisme importés signalés en Chine ont été inclus. Les variables utilisées pour comparer les cas décrits dans les anciennes zones d'endémie et les zones anciennement non-endémiques sont: l'âge, le genre, la profession, le pays visité, les espèces de *Plasmodium* et l'état de santé du patient. Les caractéristiques saisonnières et spatiales ont été analysées à partir de relevés mensuels ainsi que les distributions géographiques et spatio-temporelles. Le délai au diagnostic et à la déclaration, la méthode de diagnostic et le niveau des

établissements sanitaires impliqués ont été utilisés pour évaluer les performances de ces établissements.
Résultats : Au total, 16733 cas de paludisme, dont 90 mortels, ont été enregistrés dans 312 provinces. La majorité des cas (96,2%) ont été signalés dans des zones où le paludisme était auparavant endémique et 3,8% dans des zones où le paludisme était absent. Les patients de 19 à 59 ans et les hommes représentaient la plus forte proportion de cas dans les deux types de région. Il y avait des différences significatives entre les catégories professionnelles (p <0,001). Dans les régions anciennement impaludées, la majorité des cas concernait des travailleurs de plein air (80%). Deux pics (juin et janvier) et trois pics (juin, septembre et janvier) ont été observés respectivement dans les régions anciennement impaludées et non-impaludées. Le délai entre l'apparition des symptômes et l'admission en centre hospitalier était significativement différent entre les deux types de régions et ce pour divers types d'établissements de santé (p <0,05).
Conclusions : Toutes les zones anciennement non-impaludées signalent désormais des cas importés de paludisme. Cependant, la plus grande proportion des cas importés est observée dans les régions anciennement impaludées. Les établissements de santé situés dans les régions anciennement impaludées sont plus performants que ceux des régions anciennement non-impaludées. Une information spécifique, des antipaludiques et une surveillance doivent être fournis aux expatriés. Le renforcement des capacités et la formation continue doivent priorisés dans les établissements sanitaires en Chine.

Translated from English version into French by Prof. Roger Frutos

Импортированные случаи малярии в бывших эндемических и не связанных с марией эндемических районах Китая: существуют ли различия в характере заболевания и сроках реагирования?

Автор: Шаозен Чжан (Shaosen Zhang), Цзюнь Фэн (Jun Feng), Ли Чжан (Li Zhang), Сян Рэнь (Xiang Ren), Элизабет Джеффрой (Elizabeth Geoffroy), Сильви Мангуин (Sylvie Manguin), Роджер Фрутос (Roger Frutos), Шуйсен Чжоу (Shuisen Zhou)

Реферат

Справочная информация: В 2017 году Китай добился нулевого показателя по коренным народам. Однако наряду с политикой открытости и развитием международного сотрудничества растет число случаев завоза малярии из Китая, возвращающихся из затронутых малярией стран. Предыдущие исследования были посвящены эндемичным для малярии районам Китая. Таким образом, имеется ограниченная информация о неэндемичных районах Китая, особенно об осуществлении эпиднадзора и ответных мер в связи с малярией в медицинских учреждениях.
Методы: На основе данных регулярного эпиднадзора за малярией, собранных в период с 2013 по 2017 год, было проведено сравнительное ретроспективное исследование. Сюда были включены все случаи завоза малярии, зарегистрированные на материковой части Китая. Переменные, использованные при сравнительном анализе случаев в бывших эндемических

и бывших неэндемических районах, включали возраст, пол и род занятий, место назначения зарубежных поездок, виды плазмонии и состояние здоровья пациентов. Для сравнения сезонных и пространственных характеристик использовались ежемесячные агрегированные данные. Был проведен анализ географического распределения и пространственно-временного агрегирования. Время для диагностики и составления отчета, метод диагностики и уровень отчетности/диагностики медицинских учреждений использовались для оценки эффективности работы медицинских учреждений.

Основная часть: В общей сложности 16733 случая малярии, 90 из которых были смертельными, были зарегистрированы в 312 провинциях. Большинство случаев (96,2%) были зарегистрированы в бывших эндемичных по малярии районах, в то время как 3,8% - в бывших эндемичных по малярии районах, не затронутых малярией. Пациенты в возрастной группе от 19 до 59 лет и мужчины составляли самую высокую долю случаев заболевания в обеих областях. Существовали значительные различия между категориями профессий в этих двух областях (P<0,001). В бывших эндемичных районах наибольшая доля случаев заболевания приходилась на работников, занятых на открытом воздухе (80%). Два пика (июнь, январь) и три пика (июнь, сентябрь и январь) были обнаружены в бывших эндемических и неконтролируемых районах, соответственно. Время между началом симптомов и постановкой диагноза в клиниках было значительно разным в двух областях на разных уровнях медицинских учреждений (P<0,05).

Выводы: Все бывшие неконтролируемые районы в настоящее время сообщают о случаях завоза малярии из-за рубежа. Однако наибольшая доля случаев завоза по-прежнему приходится на бывшие эндемические районы. Медицинские учреждения в бывших эндемичных районах превзошли учреждения здравоохранения в бывших эндемичных районах. Информация, лечение и наблюдение должны предоставляться экспатриантам, а в медицинских учреждениях Китая должно обеспечиваться наращивание потенциала и постоянное непрерывное обучение.

Translated from English version into Russian by Dr. Aneta Afelt

Casos importados de malaria en China en sus áreas ex-endémicas y no endémicas: ¿existen diferencias en los perfiles de casos y tiempo de respuesta?

Autores: Shaosen Zhang, Jun Feng, Li Zhang, Xiang Ren, Elizabeth Geoffroy, Sylvie Manguin, Roger Frutos, Shuisen Zhou

Resumen

Fondo: China ha exitosamente logrado reducir à cero el numero de casos autóctonos de malaria en 2017. Sin embargo, junto con la apertura política y el desarrollo de la cooperación internacional, hay un número creciente de casos de malaria importados provenientes de ciudadanos chinos volviendo de países afectados por la malaria. Ciertos estudios previos se han centrado en áreas endémicas de malaria en China. Pero, la información existente en áreas no-endémicas de China es

muy limitada, particularmente en los reportes de los establecimientos especializados en salud y vigilancia de la malaria.
Métodos: Un estudio retrospectivo y comparativo de datos recogidos de 2013 y 2017 se llevó a cabo sobre la vigilancia de rutina de malaria. Se incluyeron casos de malaria importados y registrados en China continental. Las variables utilizadas en los análisis comparativos entre zonas antiguamente endémicas y no-endémicas, incluye la edad, el género y la ocupación, destino de viaje al extranjero, especies de *Plasmodium* y estado clínico del paciente. Los datos mensuales se han utilizado para comparar características temporales y espaciales. Se realizaron los análisis de agregación espacio-temporal y de distribución geográfica. El tiempo de obtención del diagnóstico y el tiempo de reporte, el método de diagnóstico asi como el nivel de información/diagnóstico de los establecimientos de salud fueron utilizados para evaluar la eficiencia de los establecimientos de salud.
Resultados: Un total de 16 733 casos de malaria en 312 provincias han sido registrados, de los cuales 90 han sido fatales. La mayoría de ellos (96.2%) han sido reportados en áreas anteriormente endémicas de malaria, mientras que solo 3.8% han sido reportados de áreas previamente no endémicas de malaria. La mayor proporción de casos ha sido reportada por hombres de 19 a 59 años. Se observaron diferencias significativas entre categorías profesionales en las dos áreas ($P<0.001$). En ex-áreas endémicas, la mayor proporción de casos ocurrió entre los que trabajan afuera (80%). Respectivamente, se han producido dos picos (junio, enero) y tres picos (junio, septiembre y enero) de prevalencia encontrados en áreas ex-endémicas y no ex-endémicas. El tiempo entre el inicio de los síntomas y el diagnóstico clínico ha sido significativamente diferente entre estas dos zonas con diferentes niveles de servicios de salud ($P<0.05$).
Conclusiones: Todas las áreas no-endémicas anteriores ahora están reportando casos importados de malaria. Sin embargo, la mayor proporción de casos importados aún se divulga en áreas endémicas anteriores. Los establecimientos de salud en áreas endémicas anteriores superan a los establecimientos de áreas no-endémicas. Una recomendación importante es de proporcionar información, tratamiento y vigilancia para los expatriados, así como se debe implementar el desarrollo de capacidades y de formación continua permanente en las instalaciones de salud en China.

Translated from English version into Spanish by Prof. Sylvie Manguin and Prof. Francisco Veas
